# Infiltration Patterns of Cervical Epithelial Microenvironment Cells During Carcinogenesis

**DOI:** 10.3389/fimmu.2022.888176

**Published:** 2022-07-14

**Authors:** Jianwei Zhang, Silu Meng, Xiuqing Zhang, Kang Shao, Cong Lin

**Affiliations:** ^1^College of Life Sciences, University of Chinese Academy of Sciences, Beijing, China; ^2^BGI-Shenzhen, Shenzhen, China; ^3^Guangdong Provincial Key Laboratory of Human Disease Genomics, Shenzhen Key Laboratory of Genomics, BGI-Shenzhen, Shenzhen, China; ^4^Department of Obstetrics and Gynecology, Tongji Hospital, Tongji Medical College, Huazhong University of Science and Technology, Wuhan, China

**Keywords:** cervical cancer, squamous intraepithelial lesions, microenvironment, infiltration pattern, immune response

## Abstract

**Background:**

Local cellular microenvironment plays a crucial role in the HPV-induced cervical malignant transformation. Characterization of the dynamic infiltration changes of microenvironment cells during cervical carcinogenesis would contribute to a better understanding of involved mechanisms.

**Methods:**

Three public gene expression datasets of cervical squamous epithelium samples were collected and combined. We applied seven up-to-date computational methods for infiltrating estimation and compared their results (CD4^+^ and CD8^+^ T cells) to the known fraction. After benchmarking the applied methods, the cell filtration patterns were determined and clustered through fuzzy c-means algorithm.

**Results:**

Most methods displayed better performance in predicting the abundance of CD4^+^ T cell than that of CD8^+^ T cell. The infiltration patterns of 33 microenvironment cell types (including 31 immune cells and 2 non-immune cells) were determined, and five immune cell clusters with distinct features were then derived. Meanwhile, opposite changes in abundance were observed between the activated and resting state of some immune cells from the progression perspective.

**Conclusions:**

Based on characteristics and evaluation performance of different methods, as well as previous findings, for the first time we provide a comprehensive overview of the infiltration patterns of microenvironment cells throughout cervical cancer progression.

## Introduction

Cervical cancer is thought to result from persistent infection with high-risk human papillomavirus (HR-HPV), which causes a continuum of progressive neoplastic changes known as squamous intraepithelial lesions (SILs) (1). However, precancerous lesions are not the only manifestation during cervical carcinogenesis. The cellular and acellular microenvironment surrounding the lesions undergo alterations, that also play a positive role in tumorigenesis and tumor progression (2, 3). The cellular tumor microenvironment (TME) is mainly composed of tumor vessels and perivascular cells, cancer-associated fibroblasts (CAFs), mesenchymal cells, and immune cells. Meanwhile, it is believed that non-neoplastic epithelium is also a critical component in the microenvironment of cervical lesions (4).

In premalignant lesions and cervical cancer, the host immune system (innate and adaptive immune) is closely related to HR-HPV infection. The virus induces multi mechanisms to evade immune detection, leading to chronic infection and host cell transformation (5, 6). Still, the majority of infections are spontaneously eliminated by the host immune response (7). Increasing evidence suggests that many immune cells in cervical tissue are involved in this immune response to against viruses (8). For example, NK cells could kill HPV-infected cells directly and then attract other immune cells to the sites of malignancy (9). Besides, non-immune cells are also associated with immune surveillance and disease progression (10). For example, keratinocytes synthesize various signaling and regulatory molecules (IFN-I, TNF-α and CXCL9, etc.) and antimicrobial peptides to support the recruitment and activation of immune cells (8). Hence, a better understanding of the infiltration features of microenvironment cells in the progression of the disease would lead to a new sight in the immunopathogenesis of cervical cancer.

Many traditional studies on immune cellular heterogeneity of patients with precancerous lesions have focused on peripheral blood (11, 12). The quantification of the immune infiltrates in tissue mainly relies on imaging, immunohistochemistry (IHC) and flow cytometry which generally measure a few cell subsets of interest at a time. Several current methods have been published for enumerating cell subsets proportions from gene expression profiles (13–15). This provides us with an opportunity to fully reveal the microenvironment changes across cervical disease stages. Several recent studies based on public expression profiles have described immune infiltration profiles in the preinvasive and invasive cervical lesions (16, 17). However, a comprehensive and accurate landscape of cellular microenvironment in cervical cancer progression has not been elucidated.

Herein, we performed a benchmarking between seven methods and compared their results (CD4^+^ and CD8^+^ T cells) to gold-standard measures from a meta-analysis (18). Based on the methods’ characteristics and predictive performance, we combined the estimated results of 33 cell types from various methods and constructed a landscape of cellular microenvironment transitions during cervical carcinogenesis.

## Materials and Methods

### Microarray Datasets Search and Processing

We downloaded the publicly available gene expression datasets of cervical tissue samples from Gene Expression Omnibus (GEO) database as of Oct 1st, 2021. The inclusion criteria were being no less than 25 samples, having at least three disease stages from normal, low-grade SIL (LSIL), high-grade SIL (HSIL) and squamous cell carcinoma (SCC), and being on Affymetrix platform. Three eligible microarray datasets, GSE63514 (19), GSE27678 (20) and GSE7803 (21), were collected in this study. Of note, the specimens in three studies were microdissected squamous epithelial samples. Raw CEL files of each dataset were downloaded and then preprocessed (including background correction, quantile normalization, and log2-transformation) using the Robust Multi-array Average (RMA) algorithm of the “affy” package (version 1.66.0, R foundation) (22). The datasets were combined and processed by the Combat function in the “sva” package (version 3.36.0, R foundation) (23, 24) to remove non-biological batch effects. Hierarchical clustering heatmap (Euclidean distance and Ward’s algorithm) was used to visualize correlation values between samples and detect outliers.

### Estimation of Microenvironment Cells

Computational methods, including CIBERSORTX (Relative and Absolute, web portal) (13), EPIC (version 1.1.5, R foundation) (25), ImmuCellAI (web portal) (14), MCP-counter (version 1.2.0, R foundation) (26), quanTIseq (implemented *via* the R package immunedeconv, version 2.0.4) (27, 28), TIMER2.0 (web portal) (29), xCell (version 1.1.0, R foundation) (15), were applied for microenvironment estimation. Firstly, we constructed a compendium of gene signatures collected from the tools above. Secondly, our custom probe annotation function from the in-house package was used for probes mapping. The probe mapped to multiple genes was annotated preferentially to the gene presented in the compendium. When multiple probes were mapped to the same gene, the probe with the highest mean expression was used. Finally, the results of cell composition were generated according to annotated gene expression matrix. CIBERSORTX-Relative and -Absolute scores reflect the relative fraction and absolute proportion of each cell type in a mixture, respectively. Unless otherwise specified, CIBERSORTX refers to the CIBERSORTX-Absolute score in the context.

### Analysis of CD4^+^ and CD8^+^ T Cells

We quantified CD4^+^ and CD8^+^ T cells and compared the results to the recently published formal meta-analysis (18). It should be taken into account that CIBERSORTX did not predict CD4^+^ T cell directly. Thus, the CD4^+^ T cell score was calculated as the sum of “CD4^+^ naïve T cell”, “CD4^+^ memory resting T cell”, “CD4^+^ memory activated T cell”, “T follicular helper cell (Tfh)” and “regulatory T cell (Treg)”.

CD4/CD8 ratio was also calculated. CIBERSORTX, EPIC, and quanTIseq generate relative cell fractions and allow intra-sample comparisons between cells. Other methods provide scores in arbitrary units that support inter-sample comparisons. While for xCell, we took the approach described by Marderstein et al. to calculate the ratio, adding ϵ = 10^−10^ to both the numerator and the denominator and then applying a rank-inverse normal transformation (30).

### Evaluation of Microenvironment Feature

To describe the microenvironment feature, we selected 31 immune cell types and 2 non-immune cell types for further analysis. Immune cells included 11 innate immune cell subsets [monocyte, macrophage (M0, M1 and M2), monocyte-derived dendritic cell (Mo-DC), myeloid DC (mDC), neutrophil, eosinophil, basophil, mast and natural killer (NK) cells], 3 innate-like lymphocyte subsets [natural killer T (NKT), gamma delta T (Tgd) and mucosal‐associated invariant T (MAIT) cells] and 17 adaptive immune cell subsets [CD4^+^/CD8^+^ naïve T, CD4^+^ memory T, CD4^+^/CD8^+^ central memory T (Tcm), CD4^+^/CD8^+^ effector memory T (Tem), cytotoxic T (Tc), exhausted T (Tex), Th1, Th2, Th17, Tfh, Treg, naïve B, memory B, plasma cells]. Moreover, epithelial cells and keratinocytes, as essential components of cervical epithelial microenvironment, were also incorporated.

The infiltration patterns of each cell type from seven methods were collected. Furthermore, the final pattern was determined as follows: (1) CIBERSORTX scores for activated and resting cell types were added, and the sum preferentially represented the pattern of the corresponding cell type. (2) The results of ImmuCellAI, a method for predicting comprehensive T cell subsets, preferentially represented the pattern of T cells. (3) If more than one method provides estimates for the same cell type, the pattern supported by most methods was selected. (4) If the infiltration feature of a cell type has been reported in cervical precancerous lesion or SCC (through experimental measurements), the pattern was finally corrected with reference to the previous reports (**Supplementary Table 1**).

### Fuzzy C-Means Clustering

Immune cell infiltration patterns along disease progression were clusterd using soft clustering approach of fuzzy c-means (FCM) algorithm implemented with “Mfuzz” package (version 2.48.0, R foundation) (31, 32). FCM algorithm allows each data point to belong to multiple clusters with varying degrees of membership, providing more flexibility than hard clustering. The optimal number of clusters (c) was set to five in this case. Heatmap of clusters was drawn by “pheatmap” package (version 1.0.12, R foundation).

### Statistical Analysis

R statistical software (version 4.0.3) was used for statistical analyses and graphical visualization. The Pearson correlation was used to evaluate similarity of gene expression profiles. Correlation between abundance of immune cells inferred by different methods was evaluated by Spearman correlation. Wilcoxon rank-sum test with Benjamini-Hochberg (BH) correction was applied to compare CIBERSORTX-Absolute scores. All p-values were two-sided; p < 0.05 was considered statistically significant unless otherwise specified.

## Results

### Data Integration and Quality Management

**Figure 1:**
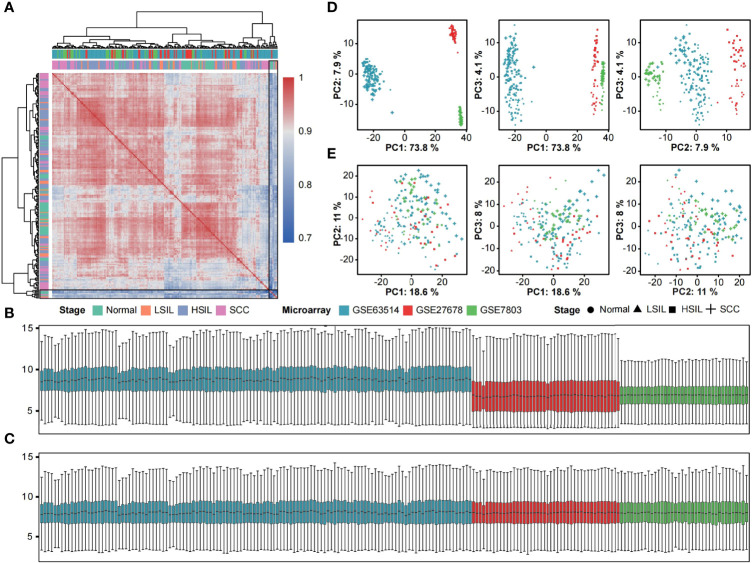
Removal of outliers and batch effect. **(A)** Heatmap displaying Pearson correlations between pairwise comparisons of meta-dataset. Clear outliers are highlighted with a black frame. Boxplots of log2-transformed gene expression distributions before **(B)** and after **(C)** batch effect correction. The boxes are colored according to datasets. Scatter plots of the first three principal components from the PCA of expression data before **(D)** and after **(E)** batch effect correction. Symbols are colored according to datasets and shaped according to disease stage.

This study merged three microarray datasets containing 46 normal, 25 LSIL, 90 HSIL and 49 SCC samples based on 13018 shared and filtered genes. A detailed description of included datasets is summarized in **Table 1**. Pearson’s correlation coefficients between samples were calculated, and a clustering heatmap was generated. Eight samples (5 normal, 1 LSIL and 2 SCC) that correlated poorly with other samples were removed from the subsequent analysis (**Figure 1A**). Next, boxplots and principal component analysis (PCA) plots were used to verify quality of gene expression data before and after batch effect correction (**Figures 1B-E**). After removing outliers and batch effect, the meta-dataset showed consistent expression distribution and reduced variance, indicating that it was suitable for further microenvironment estimation.

**Table 1:** Microarray datasets included in this study.

Dataset	Year	Platform	Country	Participants			
				Normal	LSIL	HSIL	SCC
GSE63514^a^	2015	HG-U133 Plus 2.0	USA	24	14	62	28
GSE27678^b^	2013	HG-U133A 2.0	UK	12	11	21	–
GSE7803	2007	HG-U133A	USA	10	–	7	21

a GSE63514 contains five types of samples: normal, CIN1, CIN2, CIN3 and SCC. CIN1 was defined as LSIL and CIN2/3 as HSIL.

b GSE27678 contains two platforms, and the dataset of HG-U133A 2.0 platform was analyzed.

CIN, cervical intraepithelial neoplasia.

### Benchmark of Quantification Methods

A recent systematic review and meta-analysis of infiltrating T-cell populations in cervical carcinogenesis reported fewer CD4^+^ and CD8^+^ T cells in the epithelium of SILs than in normal and SCC tissue (18). We inferred microenvironment cell types with seven computational methods and compared the estimated CD4^+^ and CD8^+^ T cells infiltration patterns to the result from meta-analysis to assess predictive accuracy of selected methods.

**Figure 2:**
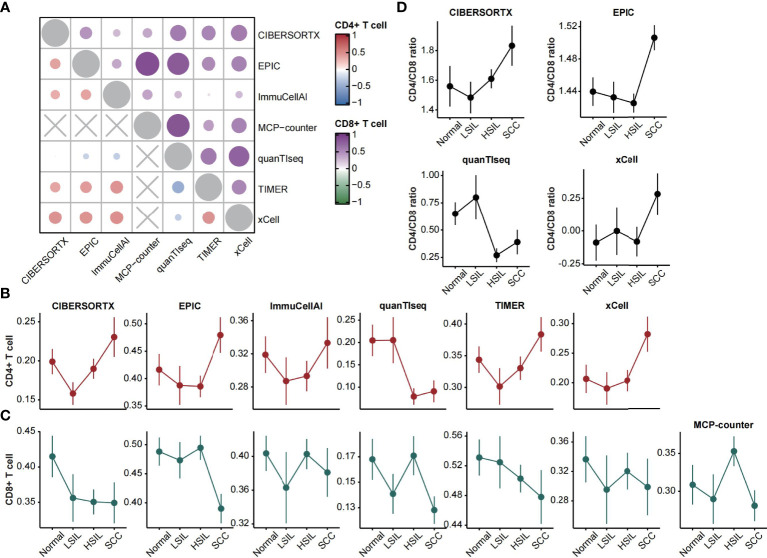
CD4^+^ and CD8^+^ T cells estimation. **(A)** Heatmap displaying average Spearman correlations of seven computational methods for the CD4^+^ (left bottom) and CD8^+^ (right top) T cells abundance across disease stages. **(B, C)** Line plots showing the abundance of CD4^+^ and CD8^+^ T cells (mean ± SEM) changes over disease stages. The estimated scores were normalized between zero and one. **(D)** Line plots showing the CD4/CD8 ratio (mean ± SEM) changes over disease stages.

We calculated the disease-stage correlation and average correlation of tested methods for the abundance of CD4^+^ and CD8^+^ T cells. In total, quanTIseq showed negative correlations with other methods in estimating CD4^+^ T cell (all Spearman’s rho < 0, **Figure 2A**). ImmuCellAI showed weak correlations with other methods in estimating CD8^+^ T cell (all Spearman’s rho < 0.4, **Figure 2A**). Moreover, the level of prediction agreement in SCC was higher than in other stages (**Supplementary Figure 1**). The analysis of CD4^+^ T cell infiltration pattern showed high similarity among methods except for quanTIseq and is consistent with the description in the meta-analysis (**Figure 2B**). In contrast, the methods’ predication on CD8^+^ T cell was inconsistent with the known pattern. Four methods (ImmuCellAI, quanTIseq, MCP-counter and xCell) showed higher CD8^+^ T cell infiltration in normal and HSIL, as well as high variances between the LSIL samples (**Figure 2C**).

Furthermore, the ratio of CD4^+^ to CD8^+^ T cells could be calculated through four feasible methods. Three of them showed high consistency and featured higher ratios in SCC. QuanTIseq showed a decreased ratio in HSIL and SCC, which corresponded to the characterization of the meta-analysis (**Figure 2D**).

### Dynamic Changes of Cellular Microenvironment During Cervical Carcinogenesis

**Figure 3:**
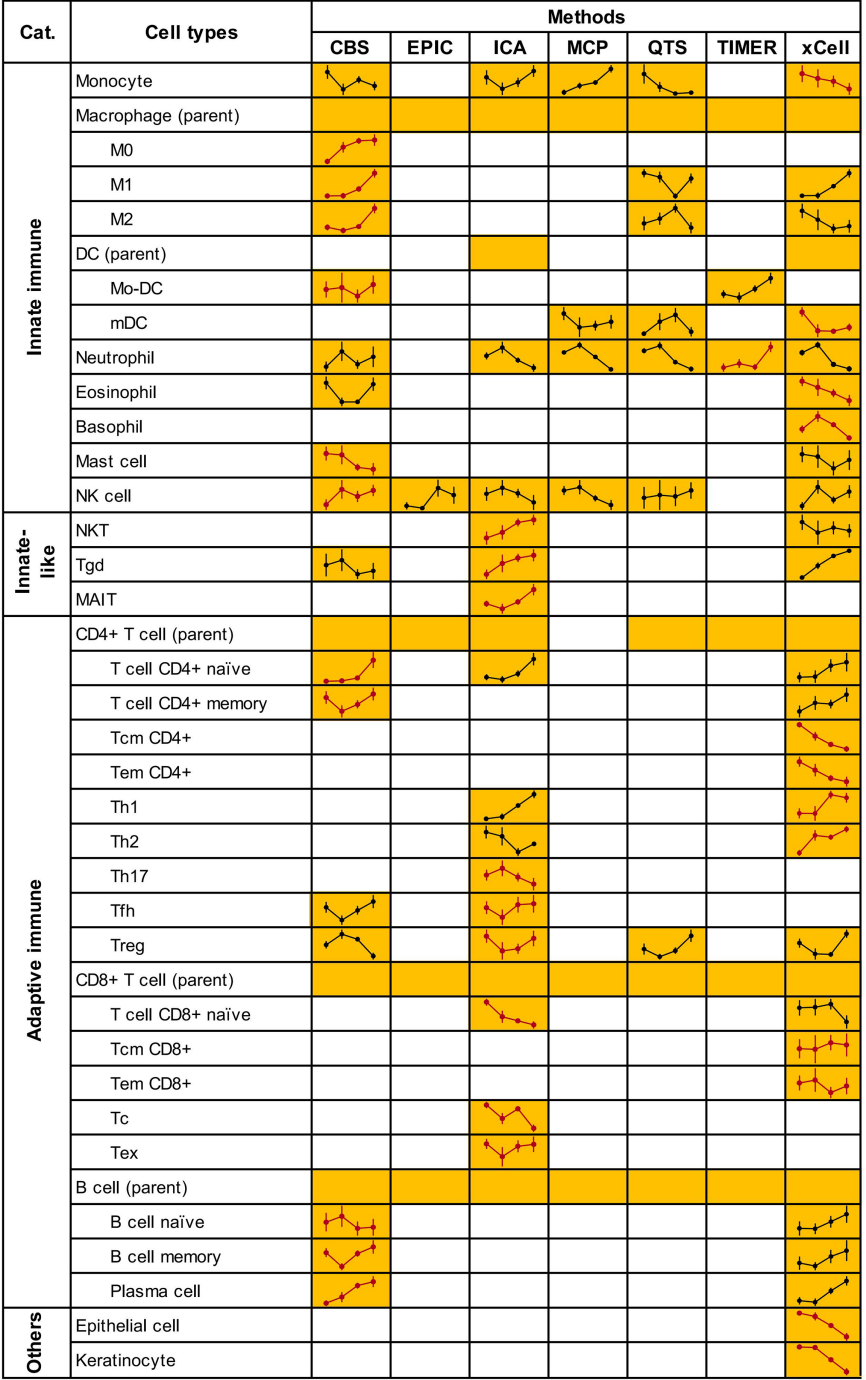
Microenvironment cells and their infiltrating patterns. Due to lack of estimation for monocyte by MCP-counter, we used the “monocytic lineage” as a surrogate. Treg score was calculated as the sum of “nTreg” and “iTreg” in ImmuCellAI. CIBERSORTX scores for activated and resting cell types (Mo-DC, mast, NK and CD4^+^ memory T cells) were also added. Yellow squares indicate that the method could predict the cells content and line plots of the predicted infiltrating patterns were added. Blank squares indicate that the method could not predict the cells content. Line plots in red represented the patterns finally determined. Full plots with normalized abundance scores of cells are shown in **Supplementary Figure 2**. CBS, CIBERSORTX; ICA, ImmuCellAI; MCP, MCP-counter; QTS, quanTIseq.

According to the variety and accuracy of predictions from applied methods, CIBERSORTX, ImmuCellAI, and xCell were selected as guidelines and the rest methods were considered as references. Specifically, the microenvironment cells and their infiltration patterns were decided preferentially by guidelines methods. We presented a set of 33 cell types, spanning 31 immune cell types from distinct subsets of innate, innate-like, and adaptive immune cells and 2 non-immune cell types (epithelial cell, keratinocyte) to reveal an evolving microenvironment with the progression of cervical lesions. Then, we enumerated these cell types available in each of the seven analytical methods. The final infiltration pattern of each cell type was determined on the basis of several criteria (see the Materials and methods section). The complete list of selected cells and corresponding estimates are shown in **Figure 3** and **Supplementary Table 2**.

**Figure 4:**
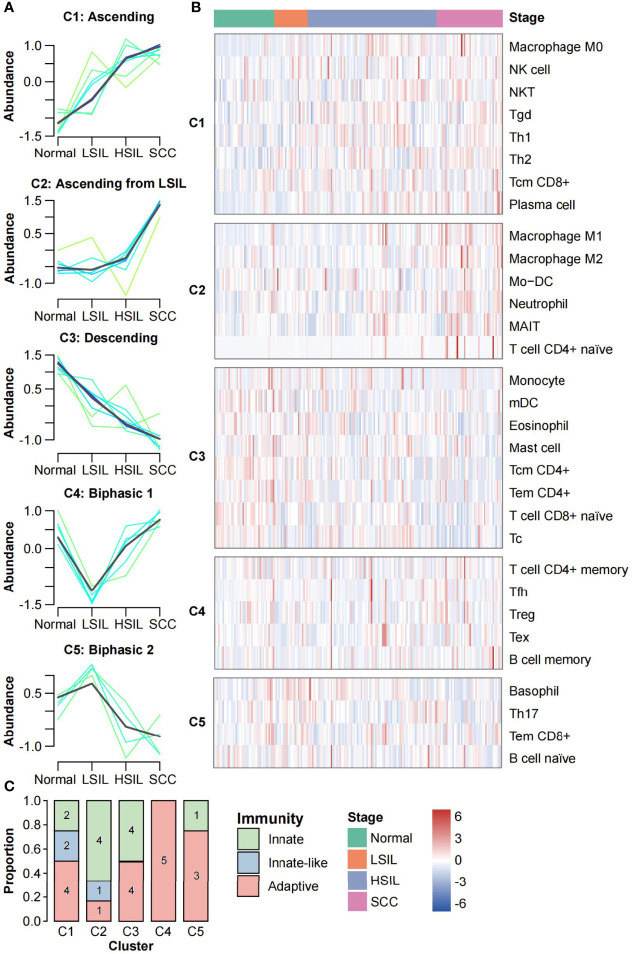
Clustering analysis of immune cells abundance. **(A)** FCM clustering identified five distinct clusters of immune cell infiltration patterns. Each line indicates the abundance of one immune cell and is colored by membership value. Green colored lines correspond to cells with low membership values. Bule and cyan colored lines correspond to cells with moderate membership values. The line for each cluster centre is plotted in black. The x axis represents disease stages; the y axis represents abundance changes. **(B)** Stage-ordered heatmap showing abundance changes of 31 immune cell types in meta-dataset. The groups were divided according to the clustering results of FCM algorithm. **(C)** Stacked bar plot of the number of immune cells in each category (innate, innate-like, and adaptive) for each cluster.

We performed the FCM clustering to characterize the dynamic changes in abundance of 31 immune cell types across the spectrum of cervical diseases. Five distinct clusters containing between 4 and 8 members per each were identified (**Figure 4A**). Clusters 1 and 3 showed linear evolution from normal to SCC (termed “Ascending” and “Descending”, respectively). Cluster 2 displayed an increased abundance from LSIL that continued to SCC; no significant difference between normal and LSIL was found (termed “Ascending from LSIL”). Two clusters had biphasic abundance evolutions; cluster 4 reached a nadir of abundance at LSIL (termed “biphasic 1”) while cluster 5 reached a peak of abundance at LSIL (termed “biphasic 2”) (**Figure 4A**). The immune cells of each cluster and their abundance changes in all samples ordered by disease progression were shown in the heatmap (**Figure 4B**). Of note, some immune cells with low membership values were not fully presented by corresponding clusters, like NK cell, Th1 cell and CD8^+^ Tcm cell in C1, Mo-DC in C2, Tc cell and mDC in C3.

We further analyzed the subsets of immune cells in each cluster. As shown in **Figure 4C**, adaptive immune cells were distributed in all five clusters, with a strong preference for C4 (n = 5, 100%) while low proportion in C2 (n = 1, 16.7%). Innate-like lymphocytes were only found in C1 and C2 (n = 2 and n = 1, respectively), showing the increase tends along the carcinogenesis. Innate immune cells were mainly distributed in C1-C3 (n = 2, n = 4 and n = 4, respectively). Interestingly, macrophages M0 were in C1, while M1 and M2 were in C2.

In addition, epithelial cells and keratinocytes showed similar infiltration patterns that the abundance slightly decreased from normal to LSIL and then dropped sharply from LSIL to SCC (**Figure 3** and **Supplementary Figure 2**).

### Evolving Immune Response During Cervical Carcinogenesis

**Figure 5:**
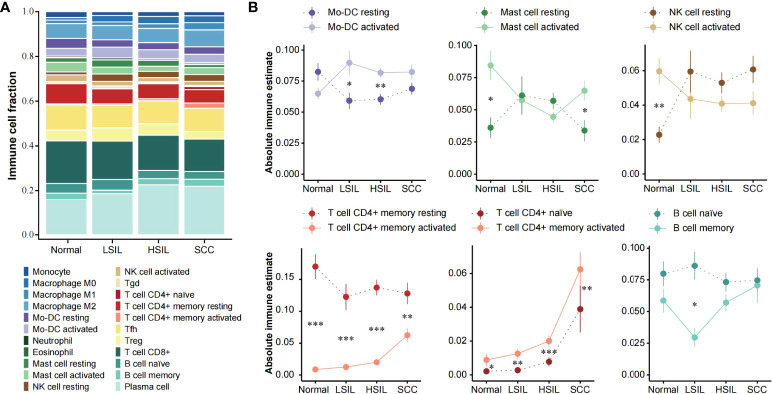
Assessment of local immune status with CIBERSORTX. **(A)** Stacked bar plot showing the composition of 22 immune cell subsets across different disease stages. The CIBERSORTX-Relative score was used. **(B)** Line plots showing immune-status shift for Mo-DC, mast cell, NK cell, CD4^+^ T cell and B cell with development of cervical lesions. Statistical comparisons were performed using Wilcoxon rank-sum test with BH false discovery rate correction. Significant differences per stage are signed with asterisks. *** p < 0.001, ** p < 0.01, * p < 0.05, FDR.

The abundance of 22 immune cell subsets of each development stage was compared using CIBERSORTX, a gene expression-based deconvolution algorithm (**Figure 5A**). Of note, CIBERSORTX could distinguish activated and resting of some cell types. Here, we explored changes in the immune status, from resting to activated and from naive to memory (**Figure 5B**).

Overall, the opposite changes in abundance existed between the activated and resting state of the same immune cell type. The alterations of activated and resting Mo-DC and NK cell abundance mainly occurred in the transition from normal to LSIL. After the relative abundance of the two states was reversed, they showed a steady infiltration from LSIL to SCC. Resting memory CD4^+^ T cell abundance was significantly higher at all stages compared to activated memory CD4^+^ T cell, but the difference between them decreased with the disease development. Interestingly, the abundance of activated mast cell decreased from normal to HSIL and then increased in SCC. Resting mast cell followed the opposite pattern and showed approximate abundance as activated mast cell in SILs. Naïve CD4^+^ T cell and activated memory CD4^+^ T cell were more abundant in SCC compared to normal and SILs. The abundance of naïve and memory B cells was basically at the same level, except for a significant separation at LSIL with a rapid decrease of memory B cell and a slight increase of naïve B cell. It also should be mentioned that all cell types discussed here were included in the microenvironment cell set.

## Discussion

The microenvironment could be characterized using either single sample gene set enrichment analysis (ssGSEA) (33) with previously defined cell signatures or published computational methods directly. The former method has an advantage in defining customized cell subsets according to our needs. Therefore, we firstly constructed a compendium of signatures related to a set of microenvironment cells from xCell, ImmuCellAI, Xiao et al.’s study (324 genes selected from CIBERSORT) (34) and Bagaev et al.’s study (35). Then, we used ssGSEA to calculate the enrichment score to represent the abundance of each cell type in each sample. We found some degree of consistency between our estimation and the original methods which the signatures were extracted from (data not shown). However, the infiltration patterns of some cells varied widely between existing methods. Hence, the estimations from ssGSEA hardly represent the full final patterns. Finally, we combined the results of multiple methods with a manual correction rather than solely depending on ssGSEA results.

It is important to reiterate that additional corrections to the estimation of bioinformatic methods based on previous studies made our conclusion more reliable. For example, recently, Wang et al. reported a decreased abundance of neutrophils from normal to SCC through bioinformatic analysis; however, the opposite results were found by flow cytometry (16). Likewise, another study also observed an increased number of neutrophils in cervical cancer compared to precursor cervical lesions (36). Consequently, we chose TIMER to describe the neutrophil pattern, which also showed the highest neutrophil infiltration in SCC, although many other methods displayed decreasing trends instead. Furthermore, Wang et al. also discovered an increased abundance of monocyte lineages as disease progressed through MCP-counter. After flow cytometry evaluation of CD14, they concluded that monocytes increasingly infiltrated in parallel along with increasing cervical lesion grade (16). In our view, flow cytometry could not reflect changes in monocytes due to CD14 being an antigen on the surface of myelomonocyte lineage rather than the monocyte itself (37). So when we evaluated monocytes, we still selected the most supported pattern from all methods instead of correcting for monocytes according to their study. Additionally, appropriate classification and combination of immune cells also contribute to the accuracy of our estimation. For example, none of methods addresses a certain DC subtype except for MCP-counter. We assigned specific subtypes to DCs estimated by different methods as reported by Sturm et al. (see their Supplementary Table 2) (28).

However, the results of some cells were inconsistent with those previously reported, such as Th17 (38), eosinophil (39) and mast cell (40) in cervical epithelium. Th17 cells in Hou et al.’s study were validated to be significantly increased in CIN3 and cervical cancer compared to healthy control through immunohistochemistry (38), while in our study, it was clustered in C5 and especially enriched in LSIL. Interestingly, a similar observation was seen in head and neck squamous cell carcinomas (HNSCC), that lymph nodes of mice with premalignant lesions showed increased Th17 cells compared to controls and HNSCC. It may be explained by the increased levels of TGF-β during progression from premalignant lesion to HNSCC, which may inhibit Th17 differentiation and decrease Th17 cells (41). Mast cells clustered in C3, on the other hand, were previously shown to progressively increase along the continuum from CIN1 to SCC (40). However, another study with fewer samples reported the least mast cell count in SCC compared to SILs and normal cervix (42), which is in line with our results. The role of mast cells in HPV lesions is uncertain. They could recruit other immune cells inducing apoptosis of tumor cells and secret IL-10 and VEGF inducing immunosuppression (43). Further studies are warranted to investigate these controversial cells and to confirm the patterns of some cell infiltrations (e.g., Tcm and Tem) firstly described by our research.

We discovered and defined five distinct clusters through clustering analysis of immune cells abundance. Extra attention should be allocated to the cells that cannot completely fit in their clusters. For example, in cluster 1, using “Ascending” to describe Th1 and Th2 cells may be inaccurate. As our results demonstrated, Th1 significantly increased from LSIL to HSIL, and slightly decreased from HSIL to SCC. The infiltration pattern of Th2 was opposite to that of Th1. The shift from the Th1 to Th2 response was found in the late stage, consistent with the previous description (44).

There are several limitations to our study. First, a few non-immune microenvironment cell subsets estimated by methods were removed in the current version for their attributes. For instance, CAFs are a group of activated fibroblasts with significant heterogeneity in the tumor stroma (45). Estimating the abundance of CAFs in squamous epithelium samples from normal cervices and SILs might be off-target. Secondly, not all studies provided the HPV status of each sample. According to the available information, most SILs+ (SILs and SCC) samples are HR-HPV positive and normal samples are a mixture of HR-HPV positive and negative samples. Regarding the impact of HPV on host microenvironment, we believe that the difference in cellular microenvironment between normal and other stage samples (especially for LSIL) has been somewhat diminished in this study (46).

## Conclusion

To conclude, we summarized the infiltrating patterns of 33 microenvironment cell types in cervical malignant transformation. Moreover, we reported a continuous shift of immune status for several typical cells. Our findings provided a reference for further studies on the mechanisms of microenvironment cells modulated by HR-HPV and corresponding immune therapies for virus clearance.

## Data Availability Statement

Publicly available datasets analyzed in this study can be found in the NCBI Gene Expression Omnibus (http://www.ncbi.nlm.nih.gov/geo/). The analysis codes supporting the conclusions of this article will be made available by the corresponding author.

## Author Contributions

Conceptualization, JZ and XZ. Methodology, JZ. Formal analysis, JZ. Visualization, JZ and SM. Writing-original draft preparation, JZ. Writing-review and editing, CL and KS. All authors have read and agreed to the published version of the manuscript.

## Funding

This research was supported by the Guangdong Provincial Key Laboratory of Human Disease Genomics (2020B1212070028).

## Conflict of Interest

Authors JZ, XZ, KS and CL were employed by company BGI-Shenzhen.

The remaining author declare that the research was conducted in the absence of any commercial or financial relationships that could be construed as a potential conflict of interest.

## Publisher’s Note

All claims expressed in this article are solely those of the authors and do not necessarily represent those of their affiliated organizations, or those of the publisher, the editors and the reviewers. Any product that may be evaluated in this article, or claim that may be made by its manufacturer, is not guaranteed or endorsed by the publisher.

## Supplementary Material

Supplementary Figure 1 Correlation matrices of methods. Heatmap displaying Spearman correlations of seven computational methods for the CD4^+^ (left bottom) and CD8^+^ (right top) T cells abundance in **(A)** normal, **(B)** LSIL, **(C)** HSIL and **(D)** SCC.

Supplementary Figure 2 The infiltration patterns of selected microenvironment cells estimated by seven methods. Line plots showing the abundance of selected microenvironment cells (mean ± SEM) changes over disease stages.

Supplementary Table 1 A collection of studies used as a reference for correction of cell infiltration patterns.

Supplementary Table 2 Normalized abundance scores of cells.
