# Supplementary figures and images for: Infiltration Patterns of Cervical Epithelial Microenvironment Cells During Carcinogenesis
Source: Front Immunol. 2022 Jul 14;13:888176. doi: 10.3389/fimmu.2022.888176 (PMC9330475; doi:10.3389/fimmu.2022.888176)

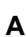

**Normal**

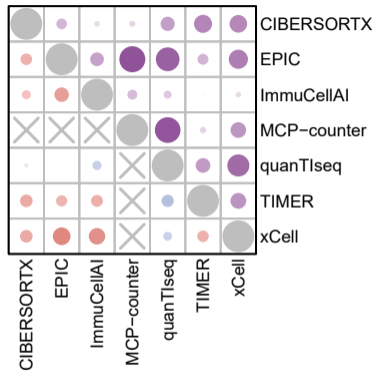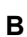

**LSIL**

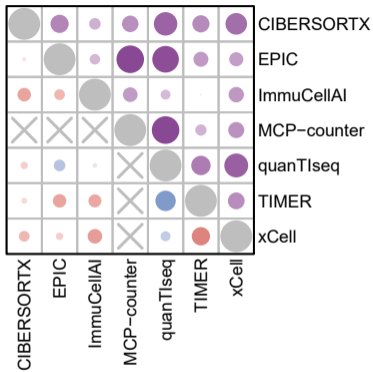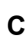

## HSIL

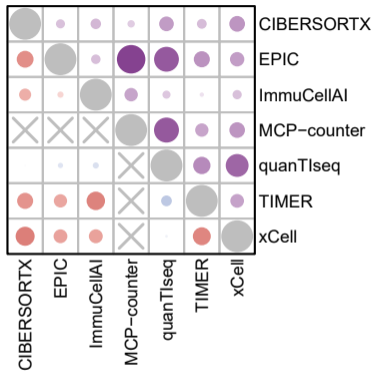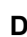

## SCC

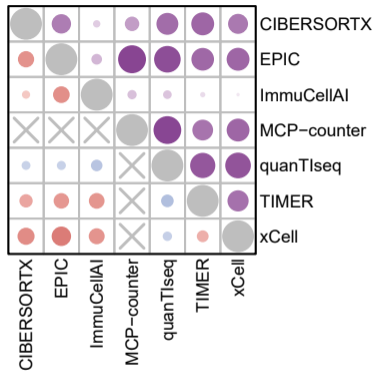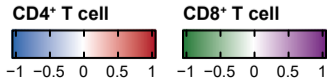

Supplement: Supplementary Figure 1 — Correlation matrices of methods. Heatmap displaying Spearman correlations of seven computational methods for the CD4+ (left bottom) and CD8+ (right top) T cells abundance in (A) normal, (B) LSIL, (C) HSIL and (D) SCC. [file Image_1.pdf]

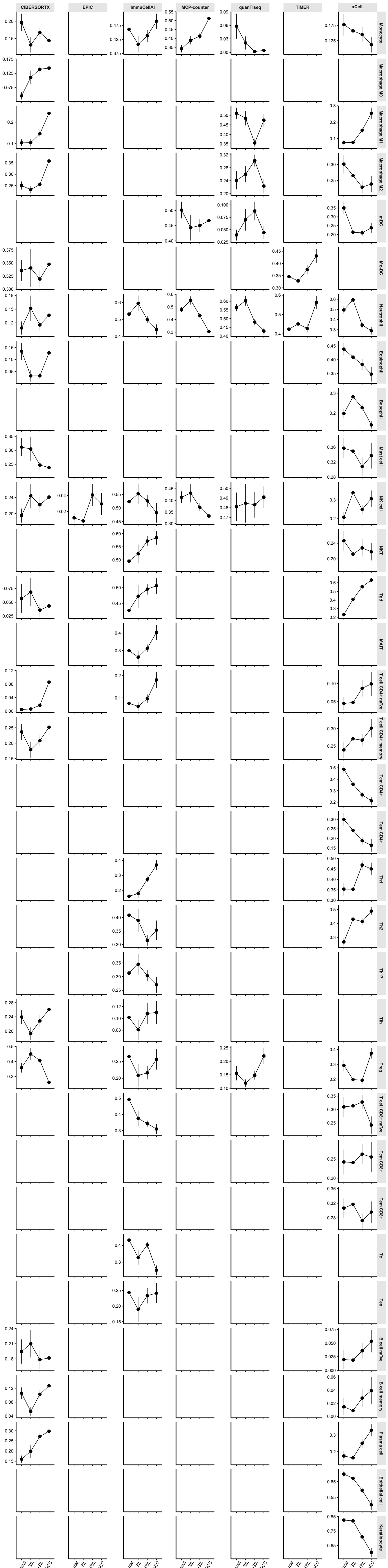

Supplement: Supplementary Figure 2 — The infiltration patterns of selected microenvironment cells estimated by seven methods. Line plots showing the abundance of selected microenvironment cells (mean ± SEM) changes over disease stages. [file Image_2.pdf]
