# Supplementary material for: Infiltration Patterns of Cervical Epithelial Microenvironment Cells During Carcinogenesis
Source: Front Immunol. 2022 Jul 14;13:888176. doi: 10.3389/fimmu.2022.888176 (PMC9330475; doi:10.3389/fimmu.2022.888176)
Supplement: Supplementary Table 1 — A collection of studies used as a reference for correction of cell infiltration patterns. [file Table_1.pdf]

Supplementary Table 1. A collection of studies used as a reference for correction of cell infiltration patterns.

Cell type	Method	Sample size	Infiltration pattern	Refs
Monocyte lineages	Flow cytometry	Total samples (N = 26): 6 normal, 6 LSIL, 7 HSIL, 7 SCC	Normal/LSIL/HSIL< SCC	(1)
Macrophage	IHC	Total samples (N = 112):26 normal 28 LSIL, 30 HSIL, 28 SCC	Normal < LSIL < HSIL < SCC	(2)
DC	IHC	Total samples (N = 45): 6 normal, 5 CIN1, 6 CIN2, 19 CIN3, 4 early SCC, 5 late SCC	Normal < CIN1 < CIN2 < CIN3<br>early SCC < CIN2/3<br>late SCC < Normal	(3)
Neutrophil	Flow cytometry	Total samples (N = 26): 6 normal, 6 LSIL, 7 HSIL, 7 SCC	Normal/LSIL/HSIL< SCC	(1)
Eosinophil	HE	Total samples (N = 60): 20 normal, 12 LSIL, 12 HSIL, 16 SCC	Normal < LSIL < HSIL < SCC	(4)
Mast cell	IHC	Total samples (N = 80): 20 normal, 13 CIN1, 15 CIN2, 17 CIN3, 15 SCC	Normal < LSIL < HSIL < SCC	(5)
	TB	Total samples (N = 45): 7 normal, 16 LSIL, 6 HSIL, 16 SCC	SCC < Normal/LSIL/HSIL	(6)
NK cell	IHC	Total samples (N = 53): 10 normal, 15 CIN1-2, 13 CIN3, 15 SCC	Normal/SCC < CIN1-3	(7)
Th17	IHC	Total samples (N = 92): 18 normal, 28 CIN3, 46 cervical cancer	Normal < CIN3 < Cervical cancer	(8)
Treg	Meta-analysis	Total studies (N = 19): 3 normal, 1 LSIL, 5 HSIL, 10 SCC	LSIL < HISL/SCC < Normal	(9)
B cell	Flow cytometry	Total samples (N = 26): 6 normal, 6 LSIL, 7 HSIL, 7 SCC	Normal/LSIL/HSIL< SCC	(1)

Abbreviations: IHC, immunohistochemistry; HE, hematoxylin and eosin; TB, toluidine blue.

References:

1. Wang Y, He M, Zhang G, Cao K, Yang M, Zhang H, et al. The immune landscape during the tumorigenesis of cervical cancer. Cancer Med. 2021;10(7):2380-95.
2. Hammes LS, Tekmal RR, Naud P, Edelweiss MI, Kirma N, Valente PT, et al. Macrophages, inflammation and risk of cervical intraepithelial neoplasia (CIN) progression--clinicopathological correlation. Gynecol Oncol. 2007;105(1):157-65.
3. Chang X, Tang J, Zhu X, Tang Y, Luo T, Li F. Immunohistochemical quantity analysis of dendritic cells and T lymphocytes in human cervical intraepithelial neoplasia and invasive carcinoma.75-80.
4. Xie F, Liu LB, Shang WQ, Chang KK, Meng YH, Mei J, et al. The infiltration and functional regulation of eosinophils induced by TSLP promote the proliferation of cervical cancer cell. Cancer Lett. 2015;364(2):106-17.
5. Jekal S-J, Lee J-A, Rho J-S. Mast cells and vascular endothelial growth factor expression in neoangiogenesis of cervical intraepithelial neoplasia and invasive squamous cell carcinomas of the uterine cervix. Korean Journal of Clinical Laboratory Science. 2005;37(3):197-206.
6. Kalyani R, Rajeshwari G. Significance of mast cells in non-neoplastic and neoplastic lesions of uterine cervix. Biomedical Research and Therapy. 2016;3(1):1-7.
7. Textor S, Dürst M, Jansen L, Accardi R, Tommasino M, Trunk MJ, et al. Activating NK cell receptor ligands are differentially expressed during progression to cervical cancer. Int J Cancer. 2008;123(10):2343-53.
8. Hou F, Li Z, Ma D, Zhang W, Zhang Y, Zhang T, et al. Distribution of Th17 cells and Foxp3-expressing T cells in tumor-infiltrating lymphocytes in patients with uterine cervical cancer. Clin Chim Acta. 2012;413(23-24):1848-54.
9. Litwin TR, Irvin SR, Chornock RL, Sahasrabuddhe VV, Stanley M, Wentzensen N. Infiltrating T-cell markers in cervical carcinogenesis: a systematic review and meta-analysis. Br J Cancer. 2021;124(4):831-41.
